# High Efficiency Direct Shoot Organogenesis from Leaf Segments of *Aerva lanata* (L.) Juss. Ex Schult by Using Thidiazuron

**DOI:** 10.1155/2014/652919

**Published:** 2014-02-04

**Authors:** K. Varutharaju, C. Soundar Raju, C. Thilip, A. Aslam, A. Shajahan

**Affiliations:** Plant Molecular Biology Laboratory, Department of Botany, Jamal Mohamed College, Tiruchirappalli 620 020, India

## Abstract

An efficient protocol for direct shoot organogenesis has been developed for the medicinal plant *Aerva lanata* (L.) Juss. ex Schult. Regeneration was achieved from leaf segments of 20 days old *in vitro* plantlets raised on Murashige and Skoog (MS) medium containing 0.25–2.0 mg L^−1^ thiadiazuron (TDZ), 3% sucrose, and 0.8% agar. After 21 days of culture incubation, maximum number of shoot organogenesis (23.6 ± 0.16) was obtained on medium containing 1.0 mg L^−1^ TDZ. The shoots were able to produce *in vitro* flowers on medium containing 1.0 mg L^−1^ TDZ in combination with 0.25–0.5 mg L^−1^ **α**-naphthaleneacetic acid (NAA). Histological observation showed that the epidermal cells of the leaf explants exhibited continuous cell division led to formation of numerous dome shaped meristematic protrusions and subsequently developed into adventitious shoots. Upon transfer of shootlets to half strength MS medium containing 1.0 mg L^−1^ indole-3-butyric acid (IBA), around 86% of the regenerated shoots formed roots and plantlets. Rooted plants were hardened and successfully established in the soil at the survival rate of 92%. The regeneration protocol developed in this study provides an important method of micropropagation of this plant. Furthermore, this protocol may be used for a large scale production of its medicinally active compounds and genetic transformations for further improvement.

## 1. Introduction

*Aerva lanata* (L.) Juss. ex Schult., a medicinal herb belonging to the family Amaranthaceae, is commonly called* Polpala*. It is endowed with various chemical compounds such as flavonoids, alkaloids, steroids, polysaccharides, tannins, phenolic compounds, and saponins [1, 2], which have contributed to its diverse uses in folklore medicine. Leaf extract of *A. lanata *is very effective in curing the urinary risk factors associated with calcium oxalate urolithiasis [3]. In addition to the traditional uses, the plant is reported for a number of pharmacological activities, namely, anthelminthic, demulcent, anti-inflammatory, diuretic, expectorant, hepatoprotective and nephroprotective, antidiabetic, antihyperglycemic, antimicrobial, cytotoxic, hypoglycemic, antihyperlipidemic, antiparasitic, and anthelminthic activities [4]. The bioactive active compounds responsible for the above pharmacological activities are *β*-carboline, *β*-sitosterol, palmitic acid, alpha amyrin, aervin, methyl aervine, and aervoside [5, 6].

The requirement of this medicinal herb is presently met from the natural populations. However, extensive utilization of this plant poses a potential threat for its existence [7]. Further, seed dormancy and seasonal availability prompted the evaluation of alternative approaches to generate the required propagation for *in vitro* studies, genetic transformation and commercial production of *A. lanata. In vitro* regeneration provides an alternative mean for large scale multiplication. Plants have been successfully regenerated through micropropagation, indirect or direct regeneration [8]. There are few reports on *in vitro* regeneration of *A. lanata*, which are also restricted to adventitious plantlet formation from shoot tip and nodal segments [7]. Direct shoot organogenesis from leaf segments represent a promising tool for mass propagation as well as genetic transformation system. To date, there is no report of direct organogenesis from leaf explants for *A. lanata.* Therefore, in the present study, an attempt has been made to develop an efficient direct regeneration system using leaf segments for *A. lanata.*

## 2. Materials and Methods

### 2.1. Establishment of Aseptic Mother Plants

Seeds of *A. lanata* were collected from basins of river Cauvery during January, 2012 and the plants were raised in the medicinal plant garden of Jamal Mohamed college, Tiruchirappalli, India. Nodal segments from *ex vitro* mother plants were used as initial explants. They were washed in running tap water for 10 min, soaked in 5% (v/v) teepol for 2 min, surface sterilized with 0.2% mercuric chloride for 10 min, and rinsed 3 times with sterile distilled water. After that, they blotted using sterilized Whatman filter paper and allowed to dry naturally. Then they were cut into small pieces of explants (0.5 cm in size) and inoculated on to MS basal media [9] supplemented with 1.0 mg L^−1^ 6-benzylamino purine BAP and 0.5 mg L^−1^ NAA. The polarity of the shoots was maintained during inoculation.

### 2.2. Culture Condition

The pH of media was adjusted 5.7 ± 0.1 before autoclaving at 121°C and 104 kPa for 15 min. All experiments were performed with semi solid media gelled with 0.8% agar powder (Himedia, Mumbai, India). Cultures were maintained at 25 ± 2°C, 16 h photoperiod under 40 *μ*mol m^−2^ s^−1^ light intensity provided by white fluorescent tubes and a relative humidity at 55–65%.

### 2.3. Influence of TDZ on Organogenesis

To study the role of TDZ on shoot organogenesis, young leaves were sectioned (approximately 0.5 × 0.5 cm) from 20 days old *in vitro* raised plants and incubated on MS medium supplemented with 0.25–2.0 mg L^−1^ TDZ for a specific period. The frequency of shoot regeneration and the number of shoots per leaf explants were recorded after 49 days of culture (starting from the initial day of inoculation).

### 2.4. Influence on *In Vitro* Flowering

To test the influence on *in vitro* flowering, shootlets from 4 weeks culture were transferred to MS basal medium containing 1.0 mg L^−1^ TDZ alone or in combination with 0-1.0 mg L^−1^ indole-3-acetic acid (IAA) or NAA.

### 2.5. Rooting and Establishment of Plantlets

For root development, 25 mm regenerated shoots were excised and cultured on half strength MS medium containing 0.5–2.0 mg L^−1^ IBA for 7 days, plantlets to pots filled with soil : perlite : vermiculate (1 : 1 : 1; v/v/v) mixture and acclimatized for 2 weeks under higher humidity before transferring to garden pots [10].

### 2.6. Histological Investigations

The origin of the adventitious shoots was studied using histological analysis. Standard procedures were followed for histological studies [11]. The samples were fixed for 24 h in FAA (70% ethanol : formalin : acetic acid = 90 : 5 : 5; v/v/v), dehydrated in a graded ethanol series, and embedded in paraffin (58°C). Sections (~10 *μ*m thick) were stained with toluene blue O. The prepared slides were examined through a light microscope (Leica, Switzerland), and all images were photographed using digital camera (Nikon, Japan).

### 2.7. Experimental Design and Statistical Analysis

All experiments were conducted using a completely randomized design and each experiment consisted of five explants per culture tube and 15 replicate tubes per treatment. Percentage of shoots producing roots and the numbers of roots formed per shoot were recorded 3 weeks after inoculation in IBA media. Data were subjected to a one way ANOVA followed by statistical significance test. The significant differences among the mean ± standard error were carried out using Duncan's multiple range tests and significance level of *P* < 0.05. (IBM SPSS ver. 19).

## 3. Results and Discussion

**Figure 1:**
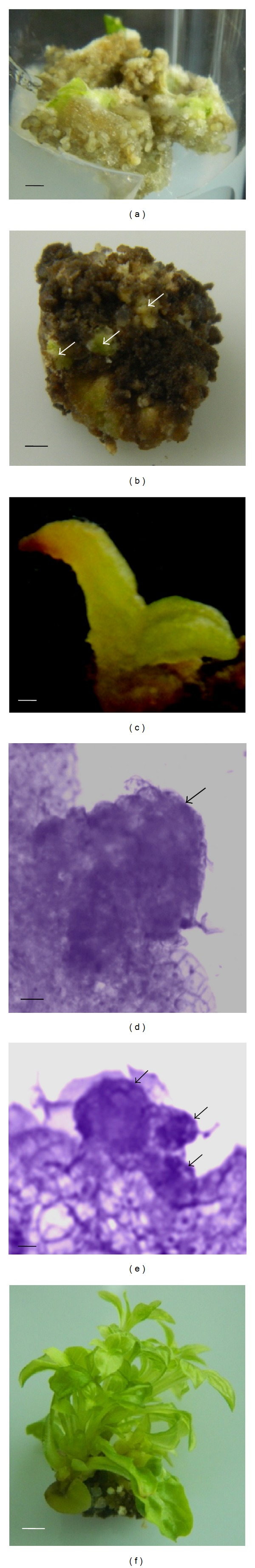
Direct organogenesis from leaf explants of *A. lanata*. (a) and (b) Initiation of adventitious shoot buds (arrowhead) from leaf explants on MS medium containing 1.0 mg L^−1^ TDZ. (c) Development of a shoot bud after 2 weeks of culture period. (d) and (e) Leaf section shows meristematic region (arrowhead). (f) Development of shoot bud after 3 weeks of culture. Bars = 2 mm ((a), (b)), 5 mm (c), 100 *μ*m ((d), (e)), and 2.5 cm (f).

Since there is no previous information on shoot development from leaf segments of *A. lanata,* there has been a new report. The effect of TDZ including concentration and duration of treatment on shoot development was initially investigated. Leaf explants were cultured on MS basal medium alone or containing various concentrations of TDZ for the induction of shoots regeneration. Leaf explants cultured in all TDZ concentrations except those in basal medium that enlarged considerably and turned green within 14 days of culture (Figures 1(a) and 1(b)). All the explants in basal medium turned brown and died within two weeks of culture. Sporadic shoot formation was observed when basal medium was enriched with TDZ (Figure 1(c)). After 28 days, more adventitious shoots were observed on leaf explants cultured on media containing 1.0 mg L^−1^ TDZ compared to the other TDZ concentrations, with an average of 23.6 ± 0.16 shoots per leaf explants and frequency of shoot regeneration of 90%. Increasing the concentration of TDZ above 1.0 mg L^−1^ resulted in a marked reduction in shoot formation in leaf explants. In the present study, low concentrations (0.25–1.0 mg L^−1^) of TDZ had a significant effect on the percentage of shoot bud regeneration from leaf segments, and higher concentration exhibited inhibitory effect (Table 1). Similar results were also reported in other plants including *Saussurea involucrata* [12] and* Solanum aculeatissimum *[13].

Consistent subculturing of the* in vitro *raised plants culture after every 4 weeks on fresh MS medium containing 1.0 mg L^−1^ TDZ led improved shoot proliferation response (Figure 1(f)). Subculture times longer or shorter than 4 weeks resulted in a decline in number of shoot bud induction. Similar results were also recorded for the medicinal plant *S. involucrata* [12], where large number of *de novo* shoots was regenerated in response to TDZ exposure and 4 weeks subculture.

It was obvious that the supplementation of TDZ in the culture media was important for direct organogenesis in *A. lanata.* Thinh [14] suggested that TDZ either increases the levels of nucleoside or the accumulation and synthesis of purine cytokinins as well as promoting the conversion of adenine to adenosine. Laloue and Pethe [15] proposed that TDZ influences the metabolism of endogenous auxins: thus altering the auxin, cytokinin ratio within the tissue, and eventually stimulating regeneration. This suggestion was also proved by several other workers [16, 17].

**Figure 2:**
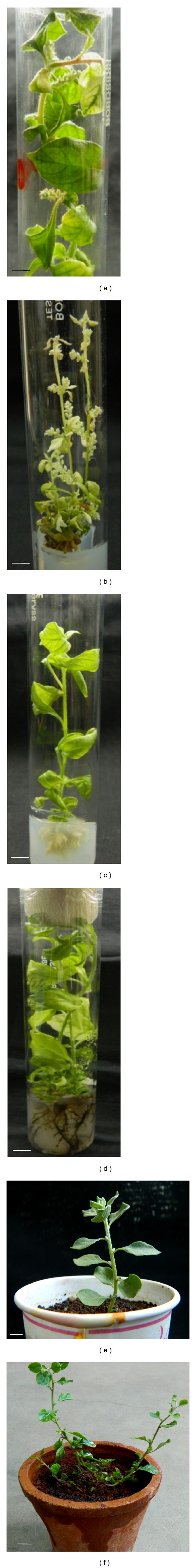
*In vitro* flowering and acclimatization of *A. lanata*. (a) *In vitro* flowering on MS medium containing 1 mg L^−1^ TDZ and 0.25 mg L^−1^ NAA. (b) *In vitro* flowering with inhibited shoot growth on MS medium containing 1 mg L^−1^ TDZ and 5 mg L^−1^ NAA. (c) and (d) *In vitro* root induction in half strength MS medium containing 1 mg L^−1^ IBA. (e) and (f) An acclimatized plant survived *ex vitro*. Bars = 3 mm ((a), (b)), 1 mm (c), 2 mm (d), and 4 mm ((e), (f)).

TDZ has been demonstrated to be effective in inducing flowering *in vitro* for several plant species [18, 19]. An interesting feature found in the present study was that the treatment of leaf explants on TDZ in combination with NAA has a positive effective on flowering *in vitro *(Figure 2(a)). Although 1.0 mg L^−1^ TDZ with 0.5 mg L^−1^ NAA achieved the highest ratio of flowering (data not shown) (Figure 2(b)), it was not suited for multiple shoot formation. Meanwhile, TDZ alone or in combination with IAA failed to induce floral bud formation. *In vitro* flowering was also observed in *Arachis hypogea* on MS medium containing cytokinins with NAA [20] and in *Withania somnifera* on MS medium containing cytokinins with IAA [21].

**Figure 3:**
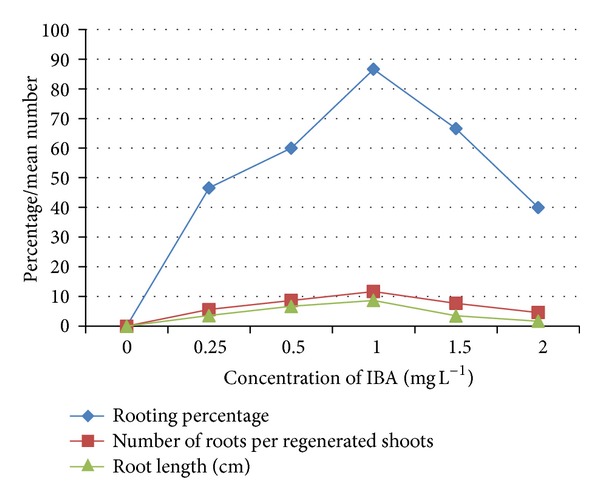
Effect of IBA on *in vitro* rooting of *A. lanata* regenerated shoots after 28 days. Data represented mean of three replicates, each with 15 cultures.

The success of micropropagation relies on the rooting percentage and survival of plantlets upon transfer to the field condition. Regenerated shoots larger than 25 mm were selected and transferred to IBA media for rooting (Figure 2(c)). The maximum frequency of rooting (86.6%) with highest number of (11.7 ± 0.15) roots per shoot was obtained in IBA at 1 mg L^−1^ after 28 days (Figures 2(d) and 3). Shoots induced by TDZ and subsequently rooted in IBA has been also reported in *Cyamopsis tetragonoloba* [22]. More than 200 plantlets with 4 to 5 fully expanded roots were successfully hardened off inside in the growth chamber within a period of 4 weeks (Figures 2(e) and 2(f)). Thereafter these plantlets were transferred to soil and were maintained in a shade house with a survival rate of 92.0%. Regenerated plants grew well and phenotypically similar to the parental stock.

Histological analysis provided morphological details of the process of organogenesis from the leaf explants of *A. lanata*. One week after culture initiation, epidermal cells of the explants exhibited continuous cell division leading to formation of numerous dome shaped meristematic protrusions with high cytoplasmic content and prominent nuclei (Figures 1(d) and 1(e)). At later stages of development, adventitious shoot formation and shoots were formed directly from these meristematic protrusions. Similar observation has also been reported for *Saintpaulia ionantha* [23], *Chirita* spp. [24], and *Titanotrichum oldhamii* [25].

## 4. Conclusion

This is the first report of direct organogenesis from leaf explants in *A. lanata.* According to the present study, TDZ is an efficient growth regulator for promoting shoot proliferation and adventitious shoot regeneration from leaf explants of *A. lanata*. However, along with NAA, it significantly influence *in vitro* flowering. Shoot proliferation rate was higher on MS medium containing 1.0 mg L^−1^ TDZ and efficient rooting was observed on half strength MS medium containing 1.0 mg L^−1^ IBA.

## Figures and Tables

**Table 1:** Effect of TDZ on shoot regeneration from leaf explants of *A. lanata. *

TDZ (mg L^−1^)	Percentage of responding explants (%)	Mean number of shoot/explants (mean ± SE)
0.0	0	0.0^f^
0.25	50	8.7 ± 0.15^d^
0.5	70	15.6 ± 0.16^b^
1.0	90	23.6 ± 0.16^a^
1.5	60	11.7 ± 0.15^c^
2.0	40	6.7 ± 0.15^e^

Data represented mean, mean ± SE (standard error) of three replicates, each with 15 cultures.

Means having the same letter in a column were not significantly different by Duncan's multiple range test (*P* < 0.05).

## Abbreviations

MS: Murashige and Skoog
TDZ: Thidiazuron
BAP: 6-Benzylamino purine
NAA: *α*-Naphthalene acetic acid
IBA: Indole-3-butyric acid
IAA: Indole-3-acetic acid.
